# Erratum

**DOI:** 10.5935/abc.20170159

**Published:** 2017-11

Edition of August 2017, vol. 109 (2), Supl. 1, p. 1-76

In the "Atualização da Diretriz Brasileira de Dislipidemias e Prevenção da Aterosclerose - 2017", published as a supplement to the *Arquivos Brasileiros de Cardiologia* [Arq Bras Cardiol 2017; 109(2Supl.1):1-76], the following corrections should be considered:

- On page 35, first paragraph, the following sentence and respective references (311 and 312) were added: "PCSK-9 inhibitors may be considered either combined with other lipid-lowering agents or isolated in statin-intolerant patients, when the targets proposed for cardiovascular risk are not met.^311,312^"
- Ref. 311: Kastelein JJ, Ginsberg HN, Langslet G, et al. ODYSSEY FH I and FH II: 78 week results with alirocumab treatment in 735 patients with heterozygous familial hypercholesterolaemia. Eur Heart J. 2015;36: 2996-3003. Ref. 312: Raal FJ, Stein EA, Dufour R, Turner T, Civeira F, Burgess L, Langslet G, Scott R, Olsson AG, Sullivan D, Hovingh GK, Cariou B, Gouni-Berthold I, Somaratne R, Bridges I, Scott R, Wasserman SM, Gaudet D; RUTHERFORD-2 Investigators. PCSK9 inhibition with evolocumab (AMG 145) in heterozygous familial hypercholesterolaemia (RUTHERFORD-2): a randomised, double-blind, placebo-controlled trial. Lancet. 2015 Jan 24;385(9965):331-40. Both references were added to the list of references, and hence the references previously numbered as 311-315 have been changed to 313-317 both in the text and in the reference list.
- On page 35, section 9.2.2.4, first paragraph, the part of the sentence "for the treatment of FH, and can be used for homozygous, as long as the defect has not been caused by a "negative receptor"" was replaced by "for FH treatment. The drug evolocumab was tested in HoFH in the Tesla B study, with an additional LDL-c reduction by 21.3%, and is not effective in homozygous forms of the disease in which the receptor is negative or nul.^318^", with inclusion of the reference 318.
- Ref. 318: Raal FJ, Honarpour N, Blom DJ, Hovingh GK, Xu F, Scott R, Wasserman SM, Stein EA; TESLA Investigators. Inhibition of PCSK9 with evolocumab in homozygous familial hypercholesterolaemia (TESLA Part B): a randomised, double-blind, placebo-controlled trial. Lancet. 2015 Jan 24;385(9965):341-50. The reference 318 was added to the list of references, and hence the references previously numbered as 316-554 have been changed to 319-557 both in the text and in the reference list.
- On page 38, section 9.2.4.2, please consider "Tangier", starting with capital letter.

